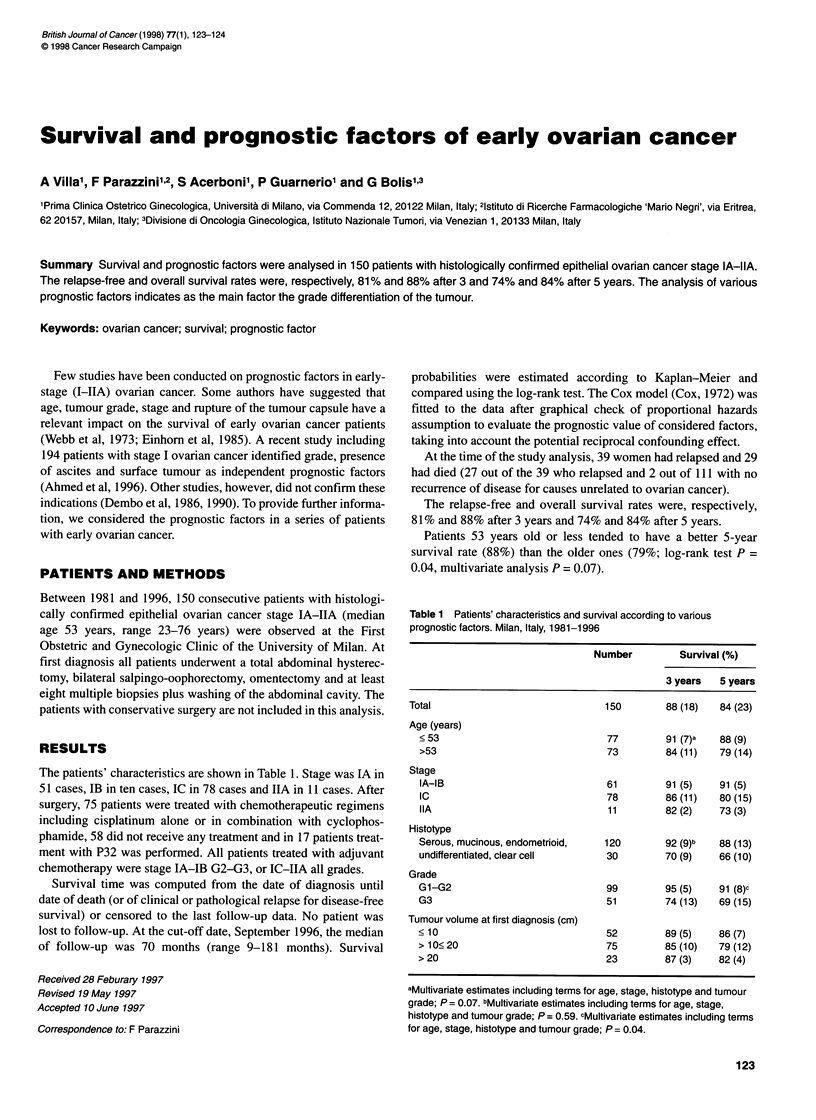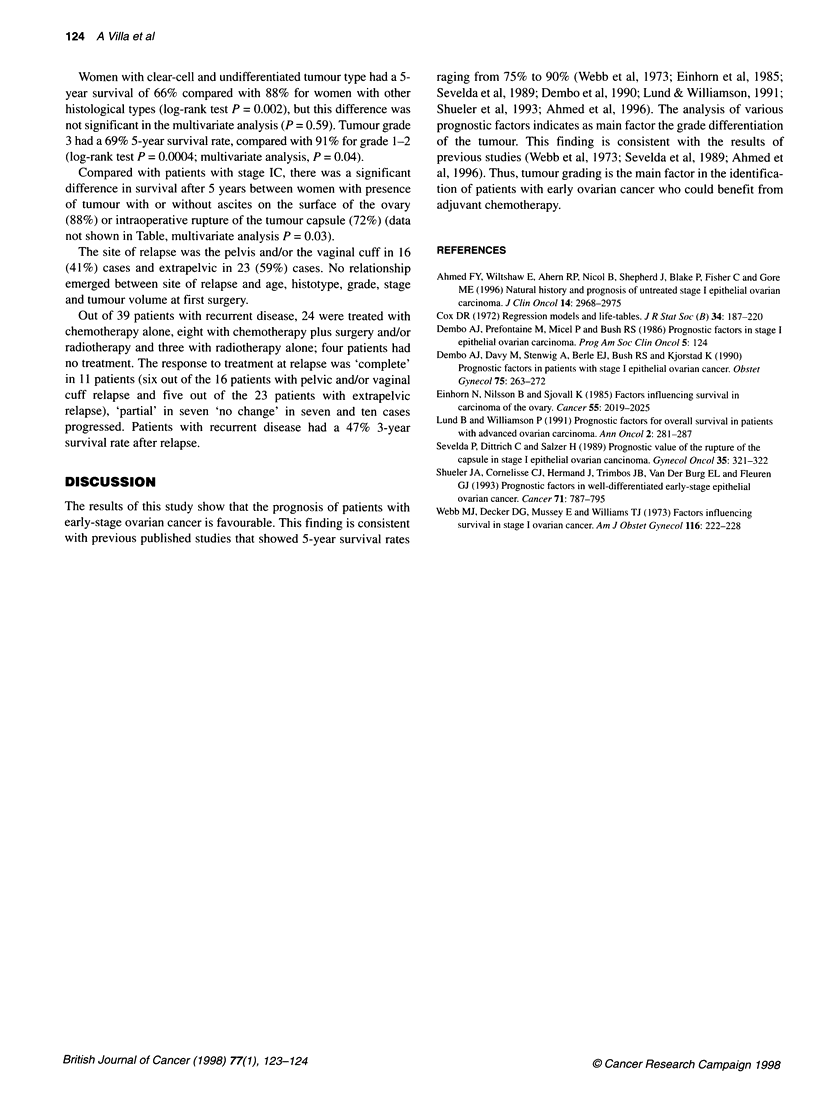# Survival and prognostic factors of early ovarian cancer.

**DOI:** 10.1038/bjc.1998.19

**Published:** 1998

**Authors:** A. Villa, F. Parazzini, S. Acerboni, P. Guarnerio, G. Bolis

**Affiliations:** Prima Clinica Ostetrico Ginecologica, UniversitÃ di Milano, Milan, Italy.

## Abstract

Survival and prognostic factors were analysed in 150 patients with histologically confirmed epithelial ovarian cancer stage IA-IIA. The relapse-free and overall survival rates were, respectively, 81% and 88% after 3 and 74% and 84% after 5 years. The analysis of various prognostic factors indicates as the main factor the grade differentiation of the tumour.


British Journal of Cancer (1998) 77(1), 123–124
© 1998 Cancer Research Campaign

### Survival and prognostic factors of early ovarian cancer

**A Villa[1], F Parazzini[1,2], S Acerboni[1], P Guarnerio[1] and G Bolis[1,3]**

[1]Prima Clinica Ostetrico Ginecologica, Università di Milano, via Commenda 12, 20122 Milan, Italy; [2]Istituto di Ricerche Farmacologiche 'Mario Negri', via Eritrea, 62 20157, Milan, Italy; [3]Divisione di Oncologia Ginecologica, Istituto Nazionale Tumori, via Venezian 1, 20133 Milan, Italy

**Summary** Survival and prognostic factors were analysed in 150 patients with histologically confirmed epithelial ovarian cancer stage IA–IIA. The relapse-free and overall survival rates were, respectively, 81% and 88% after 3 and 74% and 84% after 5 years. The analysis of various prognostic factors indicates as the main factor the grade differentiation of the tumour.

**Keywords:** ovarian cancer; survival; prognostic factor

Few studies have been conducted on prognostic factors in early-stage (I–IIA) ovarian cancer. Some authors have suggested that age, tumour grade, stage and rupture of the tumour capsule have a relevant impact on the survival of early ovarian cancer patients (Webb et al, 1973; Einhorn et al, 1985). A recent study including 194 patients with stage I ovarian cancer identified grade, presence of ascites and surface tumour as independent prognostic factors (Ahmed et al, 1996). Other studies, however, did not confirm these indications (Dembo et al, 1986, 1990). To provide further information, we considered the prognostic factors in a series of patients with early ovarian cancer.

### PATIENTS AND METHODS

Between 1981 and 1996, 150 consecutive patients with histologically confirmed epithelial ovarian cancer stage IA–IIA (median age 53 years, range 23–76 years) were observed at the First Obstetric and Gynecologic Clinic of the University of Milan. At first diagnosis all patients underwent a total abdominal hysterectomy, bilateral salpingo-oophorectomy, omentectomy and at least eight multiple biopsies plus washing of the abdominal cavity. The patients with conservative surgery are not included in this analysis.

### RESULTS

The patients' characteristics are shown in Table 1. Stage was IA in 51 cases, IB in ten cases, IC in 78 cases and IIA in 11 cases. After surgery, 75 patients were treated with chemotherapeutic regimens including cisplatinum alone or in combination with cyclophosphamide, 58 did not receive any treatment and in 17 patients treatment with P32 was performed. All patients treated with adjuvant chemotherapy were stage IA–IB G2–G3, or IC–IIA all grades.

Survival time was computed from the date of diagnosis until date of death (or of clinical or pathological relapse for disease-free survival) or censored to the last follow-up data. No patient was lost to follow-up. At the cut-off date, September 1996, the median of follow-up was 70 months (range 9–181 months). Survival probabilities were estimated according to Kaplan–Meier and compared using the log-rank test. The Cox model (Cox, 1972) was fitted to the data after graphical check of proportional hazards assumption to evaluate the prognostic value of considered factors, taking into account the potential reciprocal confounding effect.

At the time of the study analysis, 39 women had relapsed and 29 had died (27 out of the 39 who relapsed and 2 out of 111 with no recurrence of disease for causes unrelated to ovarian cancer).

The relapse-free and overall survival rates were, respectively, 81% and 88% after 3 years and 74% and 84% after 5 years.

Patients 53 years old or less tended to have a better 5-year survival rate (88%) than the older ones (79%; log-rank test $P$ = 0.04, multivariate analysis $P$ = 0.07).

**Table 1** Patients' characteristics and survival according to various prognostic factors. Milan, Italy, 1981–1996

| | Number | Survival (%) | |
|---|---|---|---|
| | | 3 years | 5 years |
| Total | 150 | 88 (18) | 84 (23) |
| Age (years) | | | |
| ≤ 53 | 77 | 91 (7)[a] | 88 (9) |
| >53 | 73 | 84 (11) | 79 (14) |
| Stage | | | |
| IA–IB | 61 | 91 (5) | 91 (5) |
| IC | 78 | 86 (11) | 80 (15) |
| IIA | 11 | 82 (2) | 73 (3) |
| Histotype | | | |
| Serous, mucinous, endometrioid, | 120 | 92 (9)[b] | 88 (13) |
| undifferentiated, clear cell | 30 | 70 (9) | 66 (10) |
| Grade | | | |
| G1–G2 | 99 | 95 (5) | 91 (8)[c] |
| G3 | 51 | 74 (13) | 69 (15) |
| Tumour volume at first diagnosis (cm) | | | |
| ≤ 10 | 52 | 89 (5) | 86 (7) |
| > 10≤ 20 | 75 | 85 (10) | 79 (12) |
| > 20 | 23 | 87 (3) | 82 (4) |

[a]Multivariate estimates including terms for age, stage, histotype and tumour grade; $P$ = 0.07. [b]Multivariate estimates including terms for age, stage, histotype and tumour grade; $P$ = 0.59. [c]Multivariate estimates including terms for age, stage, histotype and tumour grade; $P$ = 0.04.

*Received 28 Feburary 1997*
*Revised 19 May 1997*
*Accepted 10 June 1997*

*Correspondence to:* F Parazzini

Women with clear-cell and undifferentiated tumour type had a 5-year survival of 66% compared with 88% for women with other histological types (log-rank test $P = 0.002$), but this difference was not significant in the multivariate analysis ($P = 0.59$). Tumour grade 3 had a 69% 5-year survival rate, compared with 91% for grade 1–2 (log-rank test $P = 0.0004$; multivariate analysis, $P = 0.04$).

Compared with patients with stage IC, there was a significant difference in survival after 5 years between women with presence of tumour with or without ascites on the surface of the ovary (88%) or intraoperative rupture of the tumour capsule (72%) (data not shown in Table, multivariate analysis $P = 0.03$).

The site of relapse was the pelvis and/or the vaginal cuff in 16 (41%) cases and extrapelvic in 23 (59%) cases. No relationship emerged between site of relapse and age, histotype, grade, stage and tumour volume at first surgery.

Out of 39 patients with recurrent disease, 24 were treated with chemotherapy alone, eight with chemotherapy plus surgery and/or radiotherapy and three with radiotherapy alone; four patients had no treatment. The response to treatment at relapse was 'complete' in 11 patients (six out of the 16 patients with pelvic and/or vaginal cuff relapse and five out of the 23 patients with extrapelvic relapse), 'partial' in seven 'no change' in seven and ten cases progressed. Patients with recurrent disease had a 47% 3-year survival rate after relapse.

### DISCUSSION

The results of this study show that the prognosis of patients with early-stage ovarian cancer is favourable. This finding is consistent with previous published studies that showed 5-year survival rates raging from 75% to 90% (Webb et al, 1973; Einhorn et al, 1985; Sevelda et al, 1989; Dembo et al, 1990; Lund & Williamson, 1991; Shueler et al, 1993; Ahmed et al, 1996). The analysis of various prognostic factors indicates as main factor the grade differentiation of the tumour. This finding is consistent with the results of previous studies (Webb et al, 1973; Sevelda et al, 1989; Ahmed et al, 1996). Thus, tumour grading is the main factor in the identification of patients with early ovarian cancer who could benefit from adjuvant chemotherapy.

### REFERENCES

Ahmed FY, Wiltshaw E, Ahern RP, Nicol B, Shepherd J, Blake P, Fisher C and Gore ME (1996) Natural history and prognosis of untreated stage I epithelial ovarian carcinoma. *J Clin Oncol* **14**: 2968–2975

Cox DR (1972) Regression models and life-tables. *J R Stat Soc (B)* **34**: 187–220

Dembo AJ, Prefontaine M, Micel P and Bush RS (1986) Prognostic factors in stage I epithelial ovarian carcinoma. *Prog Am Soc Clin Oncol* **5**: 124

Dembo AJ, Davy M, Stenwig A, Berle EJ, Bush RS and Kjorstad K (1990) Prognostic factors in patients with stage I epithelial ovarian cancer. *Obstet Gynecol* **75**: 263–272

Einhorn N, Nilsson B and Sjovall K (1985) Factors influencing survival in carcinoma of the ovary. *Cancer* **55**: 2019–2025

Lund B and Williamson P (1991) Prognostic factors for overall survival in patients with advanced ovarian carcinoma. *Ann Oncol* **2**: 281–287

Sevelda P, Dittrich C and Salzer H (1989) Prognostic value of the rupture of the capsule in stage I epithelial ovarian cancinoma. *Gynecol Oncol* **35**: 321–322

Shueler JA, Cornelisse CJ, Hermand J, Trimbos JB, Van Der Burg EL and Fleuren GJ (1993) Prognostic factors in well-differentiated early-stage epithelial ovarian cancer. *Cancer* **71**: 787–795

Webb MJ, Decker DG, Mussey E and Williams TJ (1973) Factors influencing survival in stage I ovarian cancer. *Am J Obstet Gynecol* **116**: 222–228

*© Cancer Research Campaign 1998*